# Supplementary figures and images for: Performance-gated deliberation: A context-adapted strategy in which urgency is opportunity cost
Source: PLoS Comput Biol. 2022 May 26;18(5):e1010080. doi: 10.1371/journal.pcbi.1010080 (PMC9176815; doi:10.1371/journal.pcbi.1010080)

long (stationary) timescale

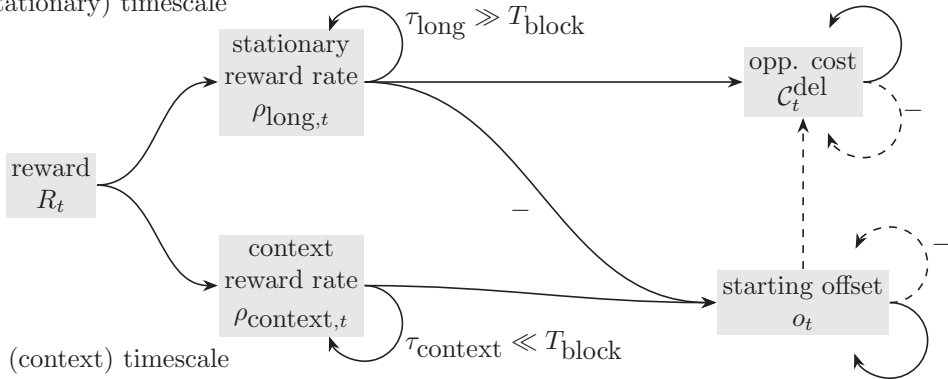

short (context) timescale

Supplement: S1 Fig — With t denoting absolute time, the reward sequence, Rt, is integrated on both a stationary (τlong) and context (τcontext) filtering timescale to produce estimates of the stationary and context-specific reward rates, respectively. These are large and small, respectively, relative to the average context switching timescale, Tblock. The estimate of the context-specific offset, ot is computed by time-integrating the difference of these two estimates. In this filtering, when a trial terminates, the effective operation is that Ctdel is set to ot, and the latter is zeroed. Thus, the opportunity cost starts at this offset and then integrates ρlong, Ct,kdel=oTk−1,k−1+ρlong,k−1t, where oTk−1, k−1 = (ρcontext,k−1 − ρlong,k−1)Tk−1. Notes on the computational graph: Arrows pass the value at each time step (dashed arrows only pass the value when a trial terminates). Links annotated with ‘−’ multiply the passed quantity by −1. (PDF) [file pcbi.1010080.s002.pdf]

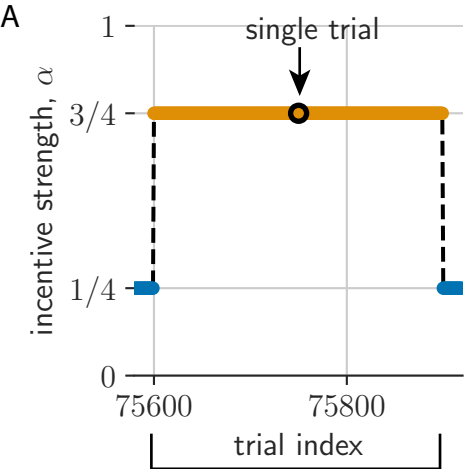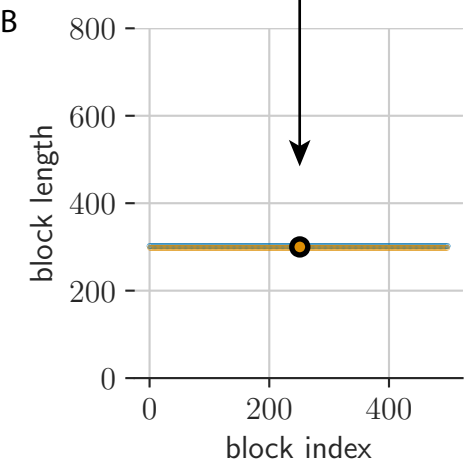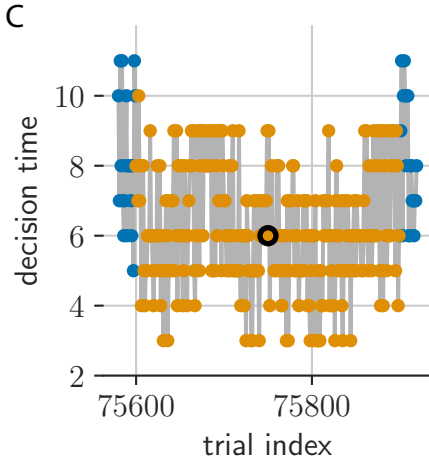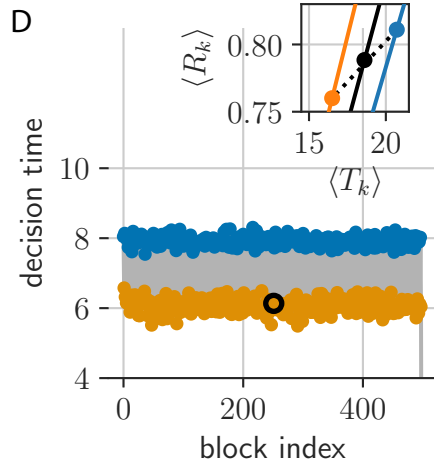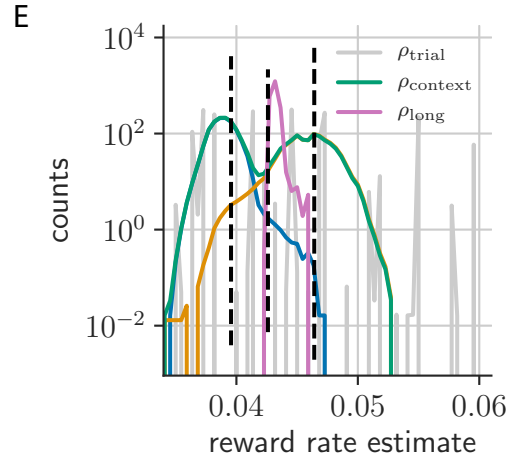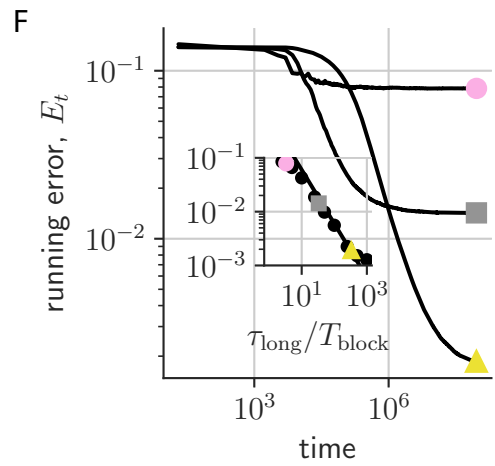

Supplement: S2 Fig — (A) Trials are grouped into alternating trial blocks of constant α (fast (orange) and slow (blue) conditions). (B) Here, trial block durations are constant over the experiment. (C) Decision times over the trials from (A) distribute widely, but relax after context switches. (D) Block-averaged decision times remain stationary. Inset shows the context-conditioned trial-averaged reward 〈Rk〉 and trial duration 〈Tk〉 (orange and blue dots; black is unconditioned average; 〈⋅〉 denotes the trial ensemble average). Lines pass through the origin (slope given by the respective reward rate). (E) Distribution of estimates have lower variance than the trial reward rates, ρtrial (gray). The conditioned averages of ρ^kτcontext shown as blue and orange. (F) The relative error in estimating ρ, Et=1t∑kt|ρ^kτlong−ρ|/ρ, for τlong = 103(circle), 104(square), 105(triangle). Inset shows that ETexp∝(τlong/Tblock)−1 over a grid of τlong and Tblock as expected (black line). (PDF) [file pcbi.1010080.s003.pdf]

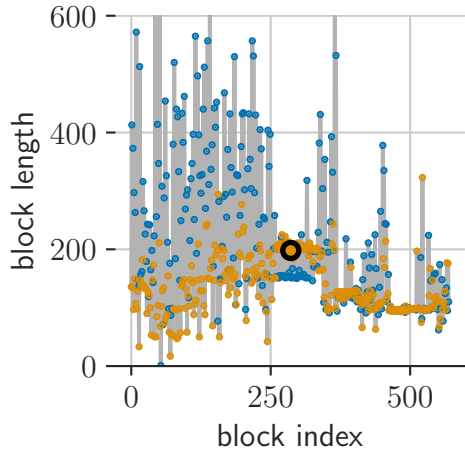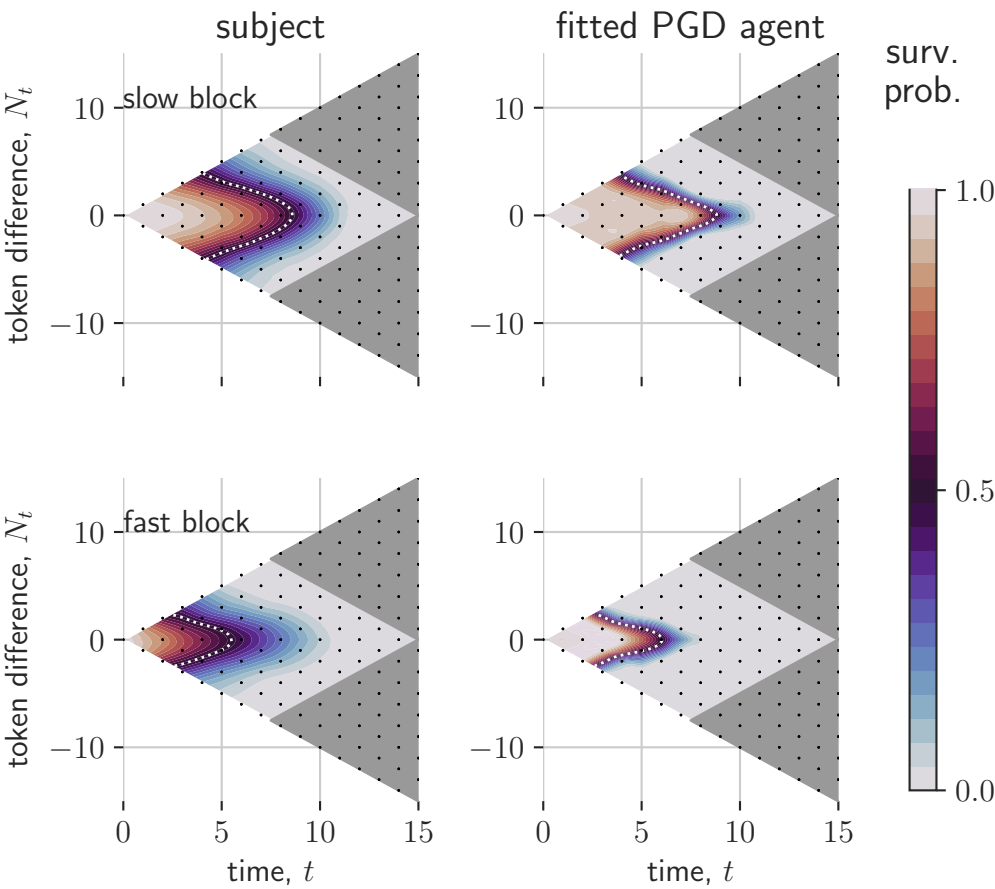

Supplement: S3 Fig — Same as Fig 5. (PDF) [file pcbi.1010080.s004.pdf]

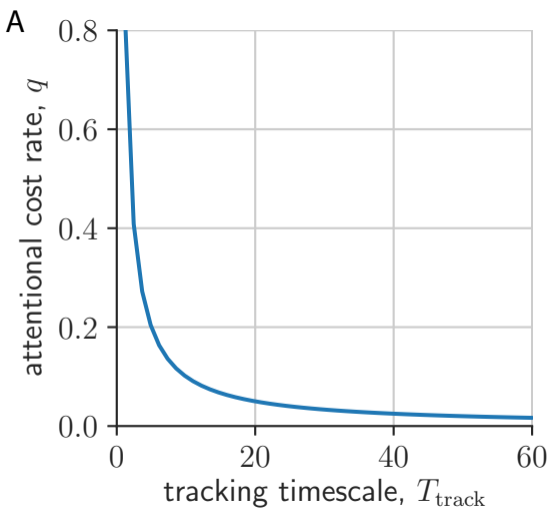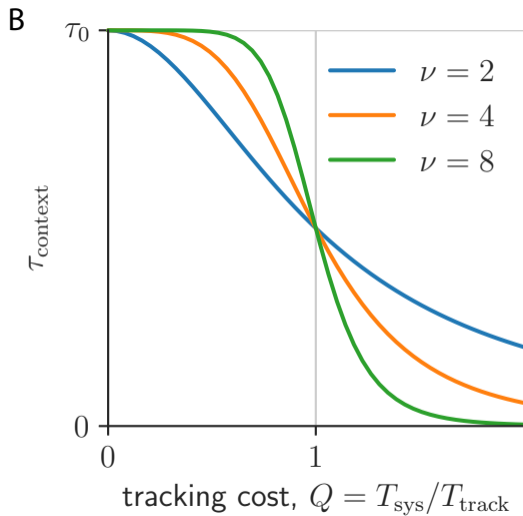

Supplement: S4 Fig — (A) Attentional cost rate, q, is set to be inversely proportional to tracking timescale, Ttrack. (B) Filtering timescale τcontext is scaled down with tracking cost, Q = Tsys/Ttrack from a base timescale, here denoted τ0 (shown for three values of sensitivity ν = 2, 4, 8). (PDF) [file pcbi.1010080.s005.pdf]

A

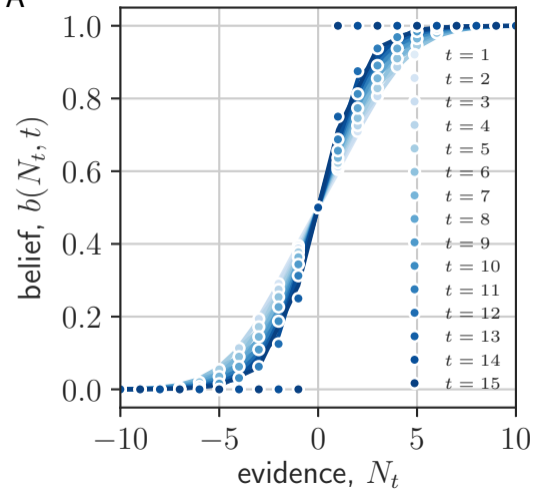

B

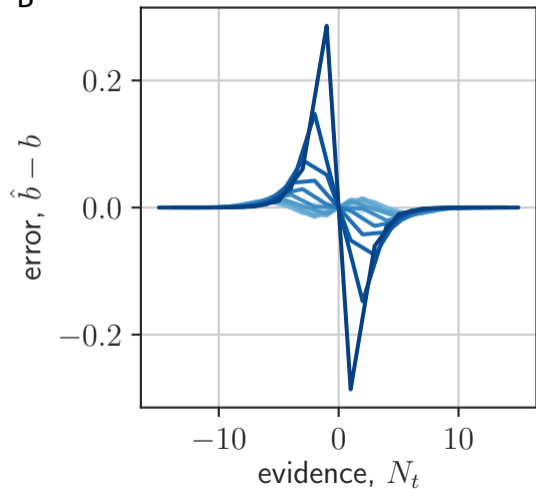

Supplement: S5 Fig — (A) the approximation explained in Methods: State-conditioned expected trial reward, for different decision times. (B) The error in the approximation for different decision times. (PDF) [file pcbi.1010080.s006.pdf]

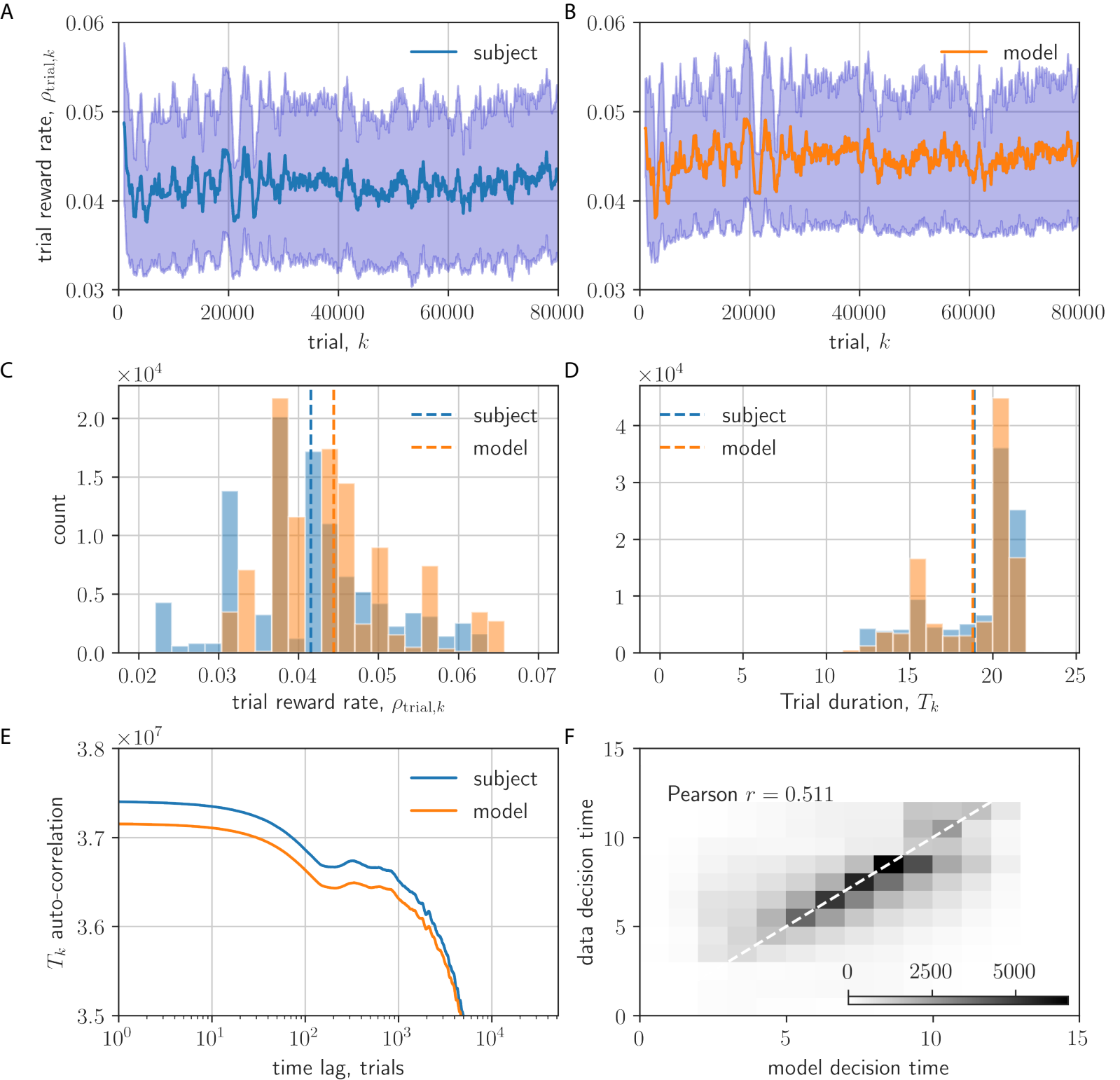

Supplement: S6 Fig — (a,b) Running average (last 1000 trial) of trial reward rate ρktrial. (c,d) Histograms of trial reward rate, ρktrial (C) and trial duration, Tk (D). (E) Auto-correlation function of trial duration. (F) Data vs. model decision time (gray-scale is count; white dashed line is perfect correlation; actual Pearson correlation is shown). (PDF) [file pcbi.1010080.s007.pdf]

A

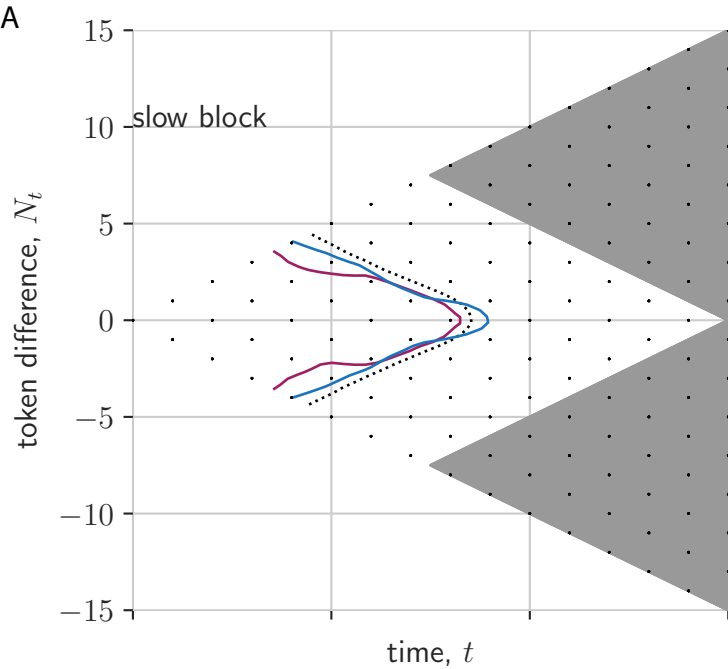

B

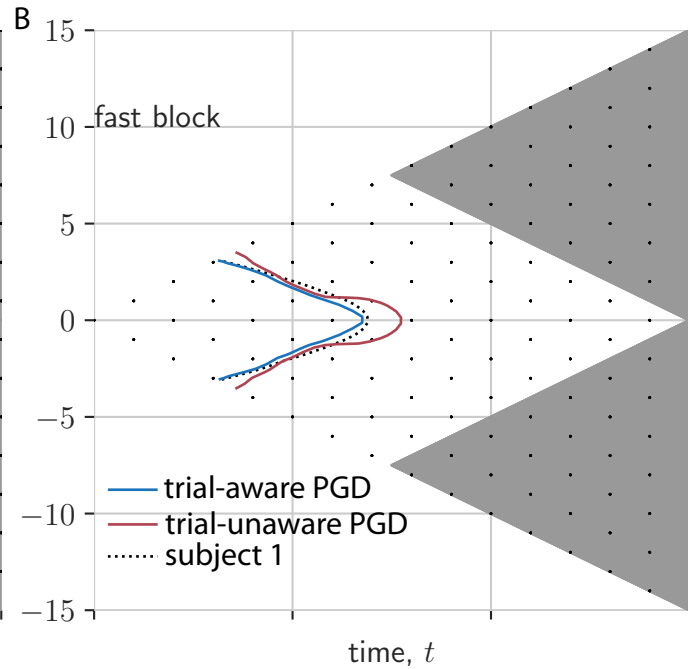

C

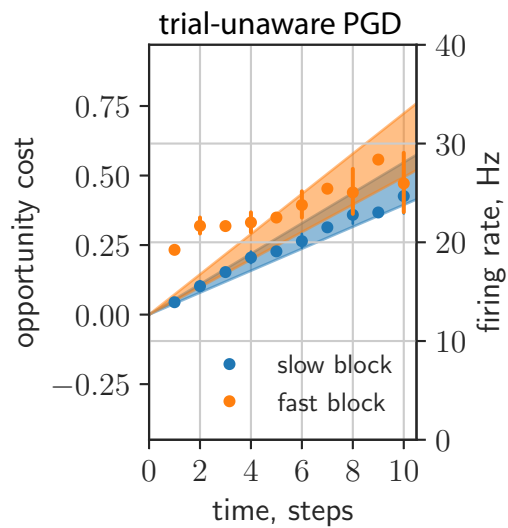

Supplement: S7 Fig — (a,b) 1/2-Survival probability contours for subject 1 (dashed), trial-aware PGD (blue), and trial-unaware PGD (red) for slow (A) and fast (B) context-conditioned data. (C) Opportunity cost for trial-unaware PGD (compare with Fig 2B). Opportunity cost range adjusted here such that data within standard error of trial-unaware PGD model prediction for slow block (blue). (PDF) [file pcbi.1010080.s008.pdf]

A

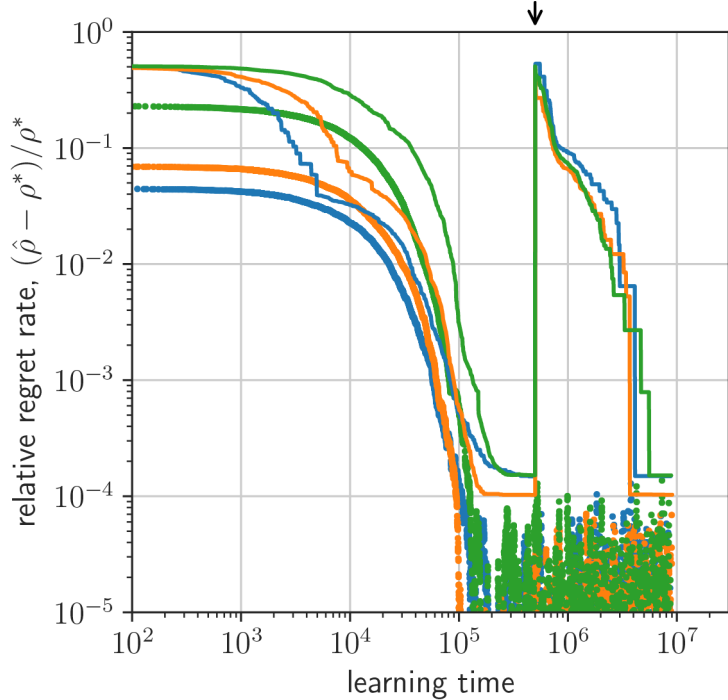

B

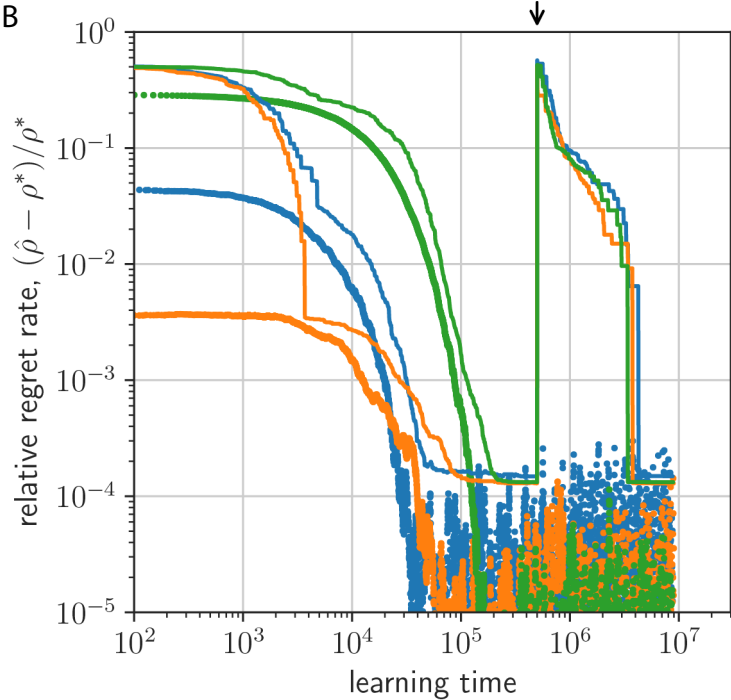

Supplement: S8 Fig — Performance is defined as relative regret rate, (ρ^−ρ*)/ρ* (PGD (dots); AR-RL (lines)). (A) Performance over different sizes of the state vector (d = 100 (blue), 200 (orange), 300 (green)). (B) Performance over different learning rates (parametrized by integration time constant, τ = 1 × 10^4^ (blue), 2 × 10^4^ (orange), 3 × 10^4^ (green)). (parameters: λ = 1/5; rmax sampled uniformily on [0, 1]). A random state label permutation is made at the time indicated by the black arrow. Values were initialized at −1. (PDF) [file pcbi.1010080.s009.pdf]

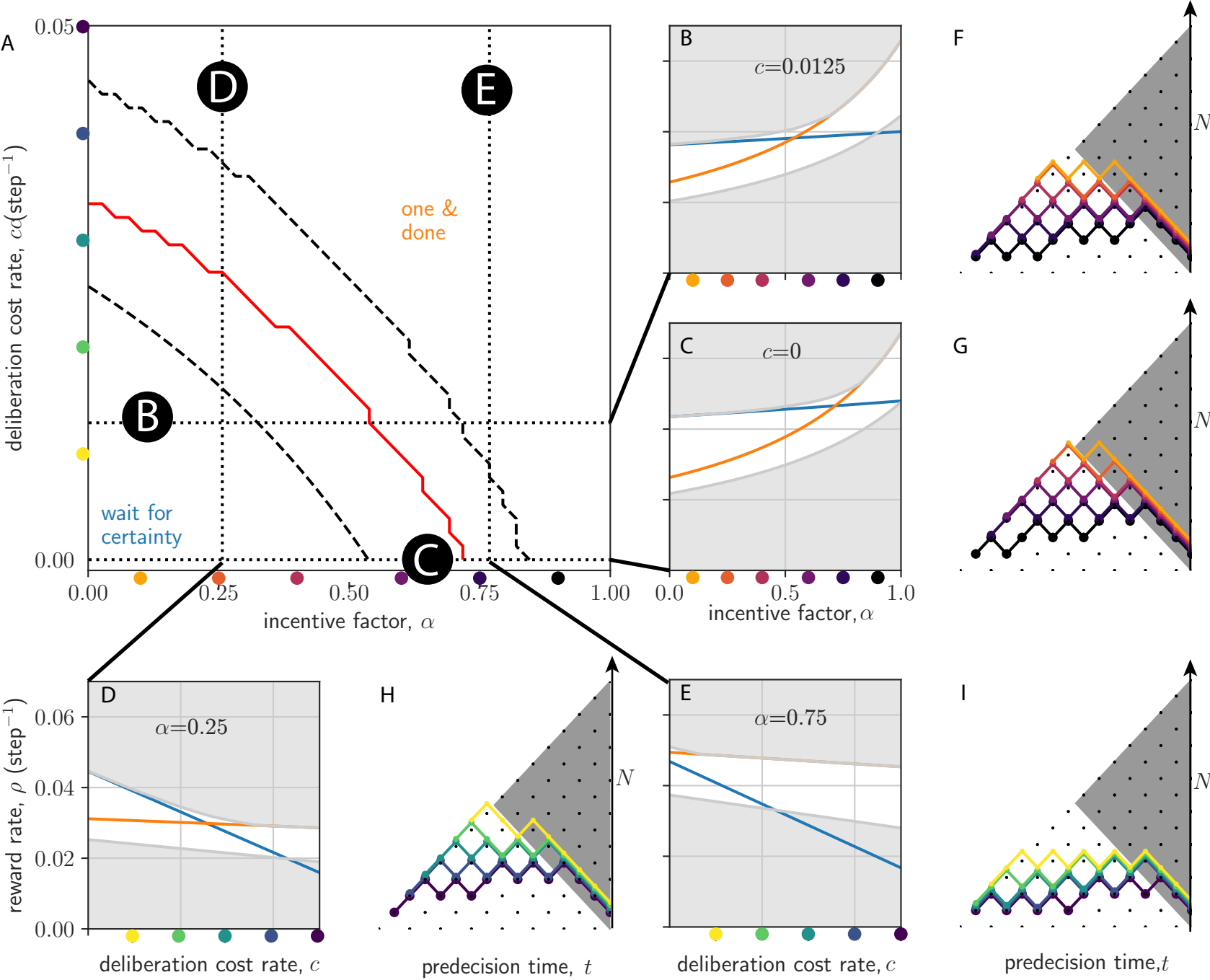

Supplement: S9 Fig — (A) The reward-rate maximizing policy interpolates from the wait-for-certainty strategy at weak incentive (low α) and low deliberation cost (low c), to the one-and-done strategy at strong incentive (high α) and high deliberation cost (high c). Dashed lines bound a transition regime between the two extreme strategies. Red line denotes where they have equal performance. (b-e) Slices of the (α, c)-plane. Shown are the reward rate as a function of α (b,c) and c (d,e) (wait-for-certainty strategy is shown in blue; one-and-done strategy is shown in orange). N is the magnitude of the token difference. (PDF) [file pcbi.1010080.s010.pdf]

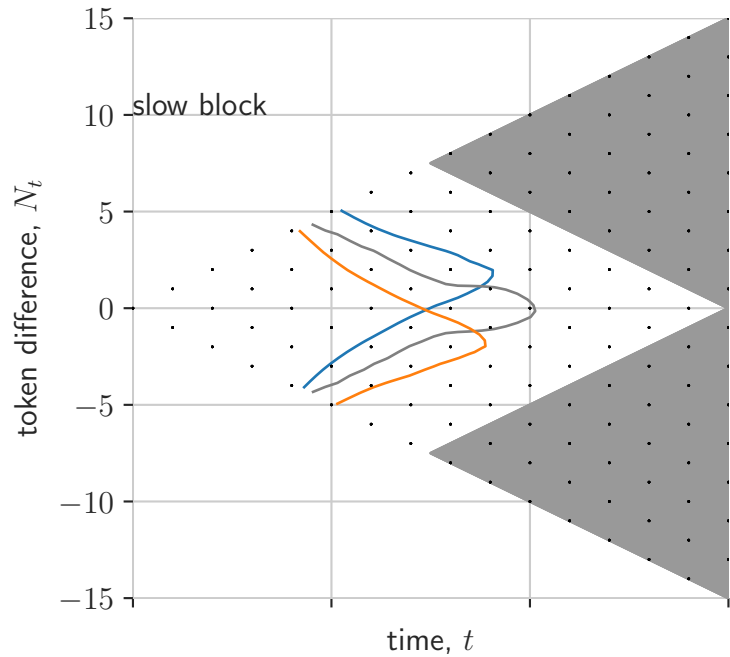

Supplement: S10 Fig — Here, we plot the half-maximum of the PGD survival probability for three values of the action reward bias, γ = −0.6, 0, 0.6 (blue, black and orange, respectively). Other model parameters same as in fitted model. (PDF) [file pcbi.1010080.s011.pdf]

Fast block

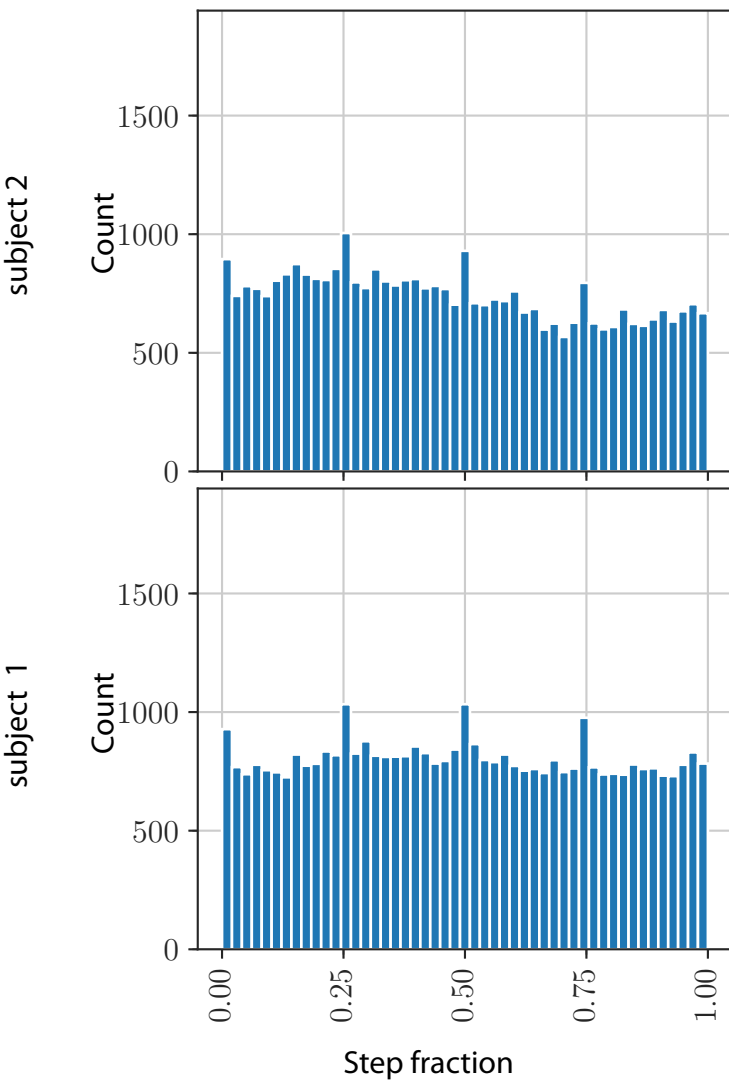

slow block

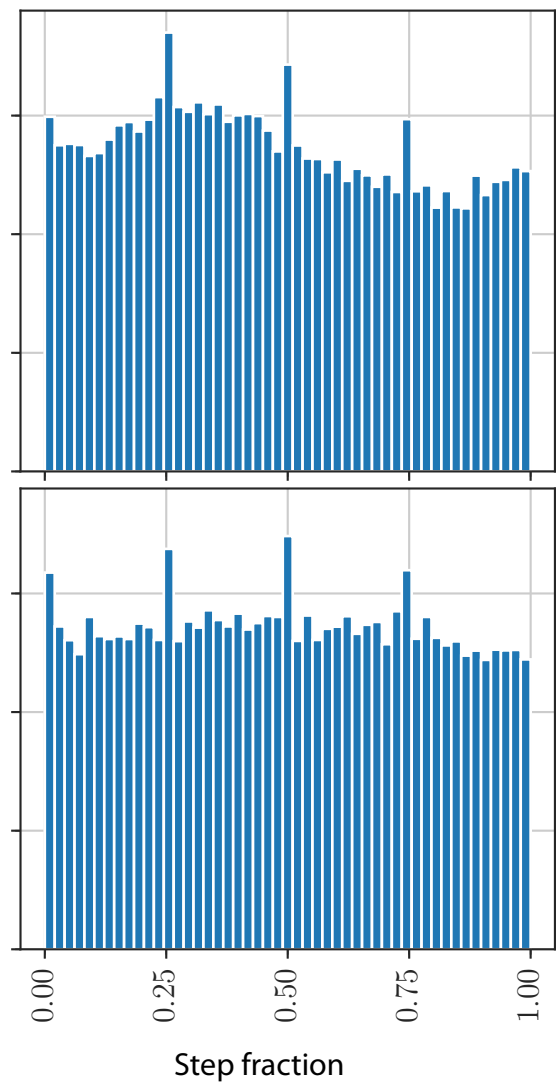

Supplement: S11 Fig — Here, we plot the histograms of decision times using their position between token jumps, the step fraction. The data is separated by α and monkey. (PDF) [file pcbi.1010080.s012.pdf]
