# Supplementary material for: Performance-gated deliberation: A context-adapted strategy in which urgency is opportunity cost
Source: PLoS Comput Biol. 2022 May 26;18(5):e1010080. doi: 10.1371/journal.pcbi.1010080 (PMC9176815; doi:10.1371/journal.pcbi.1010080)
Supplement: Attachment — Submitted filename: Untitled document.pdf [file pcbi.1010080.s013.pdf]

The question of how organisms evaluate alternatives options relative to one another as well as with respect to shifting, context dependent benchmarks in order to simultaneously optimize choice and time is unquestionably one of the most central topics of research in computational cognitive science. The authors present an impressive and well thought out model which accomodates adaptive serial comparison inspired by groundbreaking modeling work using Performance Gated Deliberation to study simultaneous alternatives.

- *"These are significant, practical complications of making decisions contingent on opportunity costs, the formal economic concept capturing the value of the alternatives lost by committing a limited resource to a given use". (line 23)*
    - Within the logical flow of the introduction, which appears to follow the customary broad-to-specific funnelling of information, it seems as though the general concept of opportunity cost should be introduced prior to the more specific discussion of the opportunity cost of time.
    - It may also be useful to unpack this concept a bit further by providing a brief example in which the limited resource with alternative uses is not time (e.g., monetary currency, energy, memory/representational capacity, or attention as later sort of touched on around line 876 where the notion of the attentional cost rate *q* is introduced).
    - Indeed, as the authors hint at, every decision between mutually exclusive alternatives implies an opportunity cost, but in the case where alternatives are presented simultaneously, the limited resource is choice itself and the opportunity cost is the value of the next best alternative. Some further discussion or explanation of opportunity costs along this line seems necessary to prepare the reader for the subsequent introduction of the two, nested opportunity costs central to the PGD model.

- *"For example, animals will learn to value a given food resource differently depending on whether it is encountered during times of plenty versus scarcity." (line 20)*
    - While it is wise of the authors to give a nod towards the optimal foraging theory early on in the introduction, it may be imprudent not to address this literature more directly via explicit citation and description. In particular, rather than simply stating *that* valuation depends on the richness of the environment, consider a brief practical demonstration of *how* valuation depends on the richness of the environment (with emphasis on *how* this relationship arises from the scarcity of time).
    - To this end, an example of patch foraging (e.g., Krebs 1974) would be particularly decorous because the manner in which this framework operationalizes the allocation of scarce time is most analogous to optimization of deliberation time in your model (compared to an example prey selection such as Krebs et al., 1978).

- "*The agent's knowledge of and ability to track context thus influences the value it assigns to possible alternatives.*"
    - Authors might add that factors that influence the perceived state value have been shown to have direct consequences on behavior.

- The notion that the correspondence between optimal movement speed and opportunity cost might account for dopamine's involvement in vigor has motivated recent work directly investigating whether a similar mechanism supports context-dependent valuation in computer-based analogues of patch foraging tasks. This work seems worth mentioning somewhere in the introduction.
    - Of particular relevance, people's willingness to leave deplenished patches have been shown to be influenced by dopaminergic depletion and replacement in Parkinson's disease (Constantino et al., 2017) and dopaminergic drugs in healthy participants (Le Heron et al., 2020).

- variance of the contextual reward distribution
    - As the proposed model accounts not just for the mean, but also the variance of reward in the local context, it would be useful to cite evidence of the behavioral relevance of this variable (e.g., Bavard et al., 2018).
    - The foraging literature also provides evidence for the role of the variance of in the contextual reward distribution (e.g., Caraco et al., 1980).

- "Performance-Gated Deliberation (PGD), that uses the increasing opportunity cost of time in a trial to collapse the decision boundary directly, by-passing the need to maximize relative value" (line 58)
    - This is only the latter part of a run-on sentence which begins in line 56, but in addition to breaking up this sentence into smaller, more palatable bites for the reader, the content of this latter part needs substantial clarification

- "The strategies for subject 2 are qualitatively similar, but shifted to earlier times relative to subject 1. Our model explains this inter-individual difference as resulting from subject 2's larger reward bias and faster context integration" (line 394)
    - This is a very interesting point and should be further unpacked and spelled out for the reader.
    - Having explained the logic behind this interpretation more comprehensively, the authors may want to point back to this result directly in the discussion with respect to hypothetical advantages of potentially examining or parameterizing inter-individual differences in future work using this sort of model.

- "The basic assumption is that tracking a signal at a higher temporal resolution should be more cognitively costly, so that adapting from faster to slower environments should happen more quickly than the reverse, so as to not pay this cost unnecessarily." (line 871)

    - I think the wording is a bit misleading here. It seems like the authors are trying to say that the model accounts for asymmetric switching costs by assuming that the temporal resolution of tracking adapts to the statistics of the environment/context and due to this, behavior is more responsive to changes from fast to slow (as opposed to the reverse) because they occur when their is greater tracking resolution.

- Out of curiosity, what do the authors propose the variable v to correspond to either cognitively or with respect to dopaminergic functioning or other mechanisms in the brain? Or rather, does v characterize something more closely related to the nature of the tracking problem which stems more innately from the task than any mechanisms in the brain.

References:

Bavard, S., Lebreton, M., Khamassi, M., Coricelli, G., & Palminteri, S. (2018). Reference-point centering and range-adaptation enhance human reinforcement learning at the cost of irrational preferences. *Nature communications*, *9*(1), 1-12.

Caraco, T., Martindale, S., & Whittam, T. S. (1980). An empirical demonstration of risk-sensitive foraging preferences. *Animal Behaviour*, *28*(3), 820-830.

Constantino, S. M., & Daw, N. D. (2015). Learning the opportunity cost of time in a patch-foraging task. *Cognitive, Affective, & Behavioral Neuroscience*, *15*(4), 837-853.

Constantino, S. M., Dalrymple, J., Gilbert, R. W., Varanese, S., Di Rocco, A., & Daw, N. D. (2017). A neural mechanism for the opportunity cost of time. bioRxiv 173443.

Krebs, J. R. (1974). Colonial nesting and social feeding as strategies for exploiting food resources in the Great Blue Heron (Ardea herodias). *Behaviour*, 99-134.

Krebs, J. R., Kacelnik, A., & Taylor, P. (1978). Test of optimal sampling by foraging great tits. *Nature*, *275*(5675), 27-31.

Le Heron, C., Kolling, N., Plant, O., Kienast, A., Janska, R., Ang, Y. S., ... & Apps, M. A. (2020). Dopamine modulates dynamic decision-making during foraging. *Journal of Neuroscience*, *40*(27), 5273-5282.
