# Supplementary material for: Performance-gated deliberation: A context-adapted strategy in which urgency is opportunity cost
Source: PLoS Comput Biol. 2022 May 26;18(5):e1010080. doi: 10.1371/journal.pcbi.1010080 (PMC9176815; doi:10.1371/journal.pcbi.1010080)
Supplement: Attachment — Submitted filename: OppCost_response.pdf [file pcbi.1010080.s014.pdf]

Dear Editor,
We would like to thank all three reviewers for their insightful reading and constructive feedback on our manuscript, which tackles a problem that Reviewer 2 thought was “unquestionably one of the most central topics of research in computational cognitive science”. We were delighted to hear that all three reviewers were enthusiastic in their support for publication, having written that our work:

- “[was] well-written and provided a convincing case for the proposed model” (Reviewer 1, hereon R1)
- “[contributes] an impressive and well thought out model” (Reviewer 2, hereon R2)
- “...makes important contributions to the current literature…” (Reviewer 3, hereon R3)

We have taken the suggestions to increase the overall clarity of the presentation by implementing all of the Reviewers’ detailed suggestions and making further edits in this direction. We have also reformatted the manuscript in the PLoS2015 latex template. This template has an Author Summary, which we have also provided. Note that we are in the process of making the github repository public so that the existing link to it in the manuscript will be active in the coming week.

Below, we provide an in-depth response to all the points raised in each review. It is formatted with indentation as:

#. Quoted reviewer comment/question

    Our response

        The quoted text we have added to the manuscript. Line numbers given are those in the manuscript version with changes highlighted.

We again thank the reviewers for their many comments. These have made the revised manuscript a much improved version.

Sincerely,

The Authors

Author Summary:

The value we place on our time impacts what we choose to do with it. Value our time too little, and we obsess over all details. Value it too much, and we rush carelessly to move on. How we value our time and how this value affects how much of it we allocate to tasks is not well-understood. The related cognitive processes are nevertheless thought to play a role in a wide range of diseases from Parkinson's to addiction. By studying this question in non-human primates, we aimed to provide a neural explanation that might serve as a foundation to studying this question in humans. We found that recorded behaviour and brain activity from a previous

experiment could be explained by a simple decision-making strategy that uses an estimate of recent performance as a way to value time. We show that this strategy explains how a brain signal called `urgency', which limits how long non-human primates deliberate, varies with context. Our work helps integrate the neuroscience of reward representations and the brain dynamics of decision-making.

Review 1

1. If I understand correctly, deliberation times should be shorter in contexts with high average reward and longer in contexts with low average reward due to the opportunity cost of time. If this is the case, it would be helpful to state this explicitly somewhere in the Introduction so that readers who are not as familiar with the theory can better understand its implications.

We agree it would be helpful. We have added the sentence in the introduction (line 50),

> “For example, it provides a normative explanation for why deliberation times are shorter in contexts with high average reward and longer in contexts with low average reward due to the opportunity cost of time.”

2. Along those same lines, how do the results of the present study relate to the finding that deliberation times in RL are shorter in contexts with higher-valued options (e.g., Fontanesi et al., 2019)? Is this something that would be predicted by PGD?

We thank R1 for bringing this reference to our attention. Fontanesi et al. 2019 present experimental results on how 4 pairs of options with stochastic rewards with overlapping distributions (with random assignment from block to block) affect the response times and accuracy of human behaviour in a bandit task paradigm. This task lacks evidence accumulation for each choice, and instead emphasizes mean reward inference from noisy rewards across trials. To the degree that we can speculate about the underlying cognitive processes at work, the response times might reflect the cognitive effort to extract the confidence of the sign of the difference of the mean rewards of this pair learned from previous trials within the block, using whatever reward representation the subject employs. A normative dependence of these response times on the total value is less clear to us. If there is a temporal integration process at work, then an opportunity cost of time is a normative means to gate response time. In this case, PGD’s increasing cost of time in a trial, with rate based on the deviation of the current context (previously estimated pair of means) should lead to the ‘magnitude effect’ found in the study whereby pairs with higher total reward, but the same difference, give shorter deliberation times. This suggests the subjects *are* using a reward representation that is storing the magnitude of the rewards as in PGD. We thank R1 for asking about the application of PGD to non-episodic tasks. This increases the breadth of interest into PGD and so we

have added the following sentence to the discussion (line 595):

> "We expect that the ideas behind PGD can also provide an explanation to the context dependence observed in response times in non-episodic tasks with varying context (e.g. in bandit tasks \cite{Fontanesi2019})"

3. Page 12, line 364: Please describe the wait-for-certainty and one-and-done strategies.

We added sentence at line 406,

> "The former decides when the success probability first hits 1 and the latter decides after a single token jump in the direction of that jump."

4. Figure 5 (B, C): Is it possible to test statistically whether the distribution of errors in slow and fast blocks differs significantly between PGD and AR-RL?

Thank you for this great question that we agree is an important and obvious check. Addressing it has made the argument for the superiority of PGD over AR-RL as a model of the data even stronger. Figure 5 (B C) shows the model error distributions of decision times for both PGD and AR-RL models. We do not have a closed form for the distribution of model decision times for either model and the empirical distributions do not appear to be well fit by simple forms such as a binomial distribution. The use of parametric tests such as a t-test to test the significance of the difference of the means of the PGD and AR-RL error distributions would be rather misguided given how non-Gaussian are their distributions. In the case of non-parametric tests, we recall that a 2-sample Kolmogorov-Smirnov test rejects the null hypothesis for a given level alpha (e.g. alpha=0.05) that the two samples (of number n and m samples) arise from the same distribution. It does so by checking whether the supremum over the domain, D_{n,m}, of the difference in the empirical cumulative distributions exceeds an alpha-dependent threshold:

$$D_{n,m} > \sqrt{-\ln\left(\tfrac{\alpha}{2}\right) \cdot \frac{1+\frac{m}{n}}{2m}}$$

In our case, there are the same number of samples between PGD and ARRL, n_slow=66897 and n_fast=36274. So, for alpha=0.05, the threshold value is 0.007 for slow, and 0.01 for fast. The supremum of the empirical distribution for slow D_slow=0.33 and for fast is D_fast=0.25. Thus, according to this test the difference in PGD and ARRL error distributions is significant in both slow and fast contexts. We have noted this significance in the text (line 418).

> "...In both contexts, PGD exhibits significantly lower error than this c^* AR-RL solution (Kolmogorov-Smirnov two-sample test)."

5. Figure 5 caption: For the survival probabilities, should it not be P(tdec > t | Nt, t) instead of P(tdec = t | Nt, t)?

Yes, this was a typo in the main text (it was correctly written in the Methods section, however). Thank you for pointing it out. It has been corrected.

6. Page 4, line 109: Can you clarify what you mean by an ensemble average? Is this just a weighted average?

By ensemble average we mean an average over all sources of randomness in the agent-environment interaction, ie. runs of the entire experiment. In our PGD simulations, randomness arises from the different sequence of possible token trajectories (i.e. random walks). Our implementation of the PGD strategy itself has no randomness, though we discuss how there are obvious places to add randomness in pursuit of better matching the variability in the behavioural data. To clarify, we have added at line 133:

"For a stochastic task environment, the definition of ρ includes an average over different realizations of the task"

Review 2

"...the general concept of opportunity cost should be introduced prior to the more specific discussion of the opportunity cost of time."

Thank you for this comment. Taking this and a similar comment from R3 as a clear indication that this difference needs clarification, we restructured the introduction and moved the definition of opportunity cost so that it precedes the opportunity cost of time. The text now reads (line 17),

{\it Opportunity cost}~\cite{Green1894} is the economics concept of the value of an alternative use that is lost when committing a limited resource to a given use. Opportunity costs are used to discount the value of allocating the resource to account for these forgone alternatives. A resource that is always limited is time. When to stop exploiting a local patch and leave to find another is central time-limited patch foraging ~\cite{Kolling2017}. More generally, how long to deliberate in sequential decision-making settings is central to a wide range of tasks….

"a brief practical demonstration of how valuation depends on the richness of the environment (with emphasis on how this relationship arises from the scarcity of time)."

We have added a sentence and the [Kolling 2017] reference here, as well as a reference regarding environmental richness (Freidin & Kacelnik, Science 2011; line 32). See the above text starting at line 17.

“Authors might add that factors that influence the perceived state value have been shown to have direct consequences on behavior.”

We have added the text below at line 33. If the reviewer had a particular reference in mind, we would be happy to include it here.

> The agent's knowledge of and ability to track context thus influences the value it assigns to possible alternatives. Factors that influence this knowledge have been shown to have direct consequences on behaviour.

“...recent work directly investigating whether a similar mechanism supports context-dependent valuation in computer-based analogues of patch foraging tasks. This work seems worth mentioning somewhere in the introduction.”

We have added the two references listed by R2 with some description. We thank R2 for [LeHeron 2020], which we had missed. At line 46,

> This perspective has been applied in clinical work through dopamine's relationship with vigor impairments. In particular, people’s willingness to leave deplenished patches have been shown to be influenced by dopaminergic depletion and restoration in Parkinson’s disease \cite{Constantino2017} and dopaminergic drugs in healthy participants \cite{LeHeron2020}.

“As the proposed model accounts not just for the mean, but also the variance of reward in the local context, it would be useful to cite evidence of the behavioral relevance of this variable (e.g., Bavard et al., 2018).
The foraging literature also provides evidence for the role of the variance of in the contextual reward distribution (e.g., Caraco et al., 1980).”

Thank you for the great suggestion. PGD would indeed be influenced by variance in reward, though the PGD decision boundary is also sculpted by the timescales chosen for the reward filters. For example, in the tokens task, there is no environmental variance in reward aside from the epistemic variability at decision time. That said, the recent flurry of work on range adaptation is a related body of work that we agree should be mentioned. We have added a sentence with these two references in the introduction at line 72:

> “For example, the variance of rewards over contexts has long been shown to factor into preferences\cite{Caraco1980} and has made its way into modern studies on the range adaptation of reward representations \cite{Bavard2018}.”

“ a run-on sentence which begins in line 56, but in addition to breaking up this sentence into smaller, more palatable bites for the reader, the content of this latter part needs substantial clarification.”

We have split up this sentence as follows on line 76,

> “We first show that value in AR-RL can be expressed using the opportunity costs of deliberation and commitment. Here, the commitment cost is the shortfall in reward (relative to the maximum possible in a trial) that is expected to be lost when committing to a decision at a given time.”

and added the clarification afterwards,

> “The deliberation cost integrates the estimated cost rate of time.”

“...[Subject-specific parameter comparisons] is a very interesting point and should be further unpacked and spelled out for the reader.”

We have rewritten this part to specify the exact relationships between the variables that we refer to here and have added the following to the discussion at line 600,

> “When fit to recorded behaviour from each subject across a set of subjects, our model can be used in clinical contexts to dissect inter-individual differences via the differences in the fitted model parameters. We gave an example here for the two non-human primates, explaining the significantly smaller mean response time of the second subject as due to higher reward bias and shorter memory.”

“ (line 871) I think the wording is a bit misleading here. It seems like the authors are trying to say that the model accounts for asymmetric switching costs by assuming that the temporal resolution of tracking adapts to the statistics of the environment/context and due to this, behavior is more responsive to changes from fast to slow (as opposed to the reverse) because they occur when their is greater tracking resolution.

Thanks for the comment. We will further clarify this. Indeed, the integration time adapts to smaller values when resolution mismatch costs are higher. These costs are higher after switching from a fast to a slow context relative to the reverse. We believe the sentence as stated correctly reflects what the model does. Nevertheless, we restated it on line 956 as follows, hoping it better conveys the idea,

> “A cost-aware strategy addressing this fact is to adapt the integration time to smaller values when resolution mismatch costs are higher so the system lowers

> this higher cost quickly. For the tokens task, adapting from faster to slower environments should then happen more quickly than the reverse.“

“ what do the authors propose the variable v to correspond to either cognitively or with respect to dopaminergic functioning or other mechanisms in the brain? Or rather, does v characterize something more closely related to the nature of the tracking problem which stems more innately from the task than any mechanisms in the brain.”

> Thank you for the great question. For any task, the PGD model strategy has a component that tracks context-specific performance variation. This tracking is implemented by averaging recent history according to a set timescale. We assume the subject sets this timescale parameter to lie in a range that captures the observed variation. This solution does not require a context-specific representation, which is an advantage of the strategy that we discuss in the foraging example earlier in the main text. It is not obvious at first that such a context-free model would be able to fit well data that presents asymmetric relaxation times after context switches. However, we showed that augmenting the context-free model with a normative cost model is in fact able to fit such data and still avoids the use of a context representation. In this normative model, the tracking sensitivity parameter, \nu, is the sensitivity of the subject to this cost in a similar sense by which humans exhibit loss aversion. We interpret it as the meta-cognitive belief of the cognitive effort the subject expends in its performance tracking. Meta-cognitive beliefs have been studied extensively in cognitive neuroscience, typically associated with dorsomedial prefrontal and bilateral orbitofrontal cortex, but the details of their neural substrates are not yet well-established and we do not have an explicit proposal here. We have added at line 972:
>
> > “We note that ν captures the meta-cognitive belief that the subject has about how cognitively effortful is its tracking behaviour.”

<u>Review 3</u>

A…. I would suggest at least explaining the token task earlier in the manuscript (perhaps by adding a figure, but also explain what are the contexts and what is the learning aspect of this task). Also, AR-RL might not be super obvious/familiar to most readers and it could be better introduced.

> We appreciate the reviewer’s concern as to clarity of the presentation. We originally chose to delay introducing the tokens task until after the presentation of the PGD strategy because PGD is the main contribution of the paper and the tokens task is an application. This is a standard choice that we believe allows the reader to appreciate PGD’s general applicability, before getting into the details of the application. We had also included a schematic of the tokens task in the submitted manuscript as a figure panel

(4A), and a figure of the overall paradigm in figure 1. That said, we agree with R3 that the manuscript could be more descriptive of what context means and what the subject needs to learn to perform well. We have added sentences to this effect when we introduce the tokens task at line 310:

> The subject is thus tasked with learning the statistics of the number of tokens at the end of the trial conditioned on an intermediate state in order to balance accuracy and deciding early.

As for improving the clarity in how we introduce AR-RL, we have removed a cumbersome inline equation and made the remaining text more concise. Starting at line 146:

> AR-RL centers around the average-adjusted future return, which penalizes the passage of time by using the average reward as a cost rate. Value is defined as this future return averaged over trial statistics. This average of a sum into the future of reward deviations from the average converges without a discount factor on account of the transient effects of conditioning the statistics on the state at which the decision is made. The AR-RL algorithms we consider aim to achieve the highest ρ by also maximizing the average-adjusted value (see \hyperref[sec:epiDM]{Methods} for details).

B. The authors studied incentivized decisions where the time spent on the decision itself is a missed opportunity value, depending on the context. I wondered whether
1) it can be shown that subjects learn this across trials (with analyses or a figure);

Thank you for the great question. Indeed, what mainly needs to be learned in PGD is a timescale over which the context-specific performance can be estimated in a way that roughly solves the bias-variance problem dependent on the fixed context switching interval (this timescale determines the value of the time spent). We have specified the PGD model here to use a single value for this timescale obtained from the best fit from the data. Thus, the model ignores the early period during which this timescale is learned as well as the non-stationarity in the switching interval that presumably would contribute to ongoing learning of this timescale (see fig4a;Sfig3a).

With regards to this early period, the behavioural training protocol involved trials that built up to the complexity of the tokens task, beginning with trials that simply had a single token jump. The animal subjects were presented with trial blocks with duration that was initially fixed, but then varied. Current experiments in the Cisek lab are being conducted that more finely track this early learning portion of task training and we will be considering PGD as a model with which to analyze that data. As for the data we have available to us from Thura et al. J. Neuro 2014, which begins as the subjects first start performing full versions of the task, there are subject-specific signatures of some

learning in this initial phase. For example, we copy here Fig.2a from Thura et al. J. Neuro. (2014):

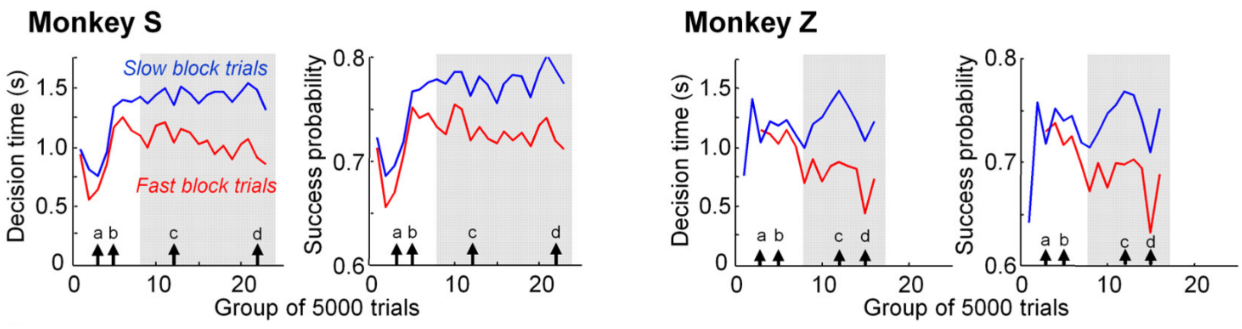

2) there are any individual differences in this learning process that could be modeled?

As mentioned in the previous paragraph and figure, there is subject-specific learning. Given the opportunity to design an experiment to capture this learning process, we do believe various models could be tested and meaningful individual differences extracted. We leave this for future work.

C.This brings me to my doubts about the chosen sample: Are these 2 monkeys representative of the behavior of other monkeys? And of humans as well? Figure 5A compares the 2 monkeys' behavior with a "reference expert human". Citation 31 also specifies "Single subject behavioural data shared by Thomas Thierry." This should be better explained. Is this published data? How representative are these data of human behavior?

Thank you for this question. We agree that the question of representativeness is important and we provide new analysis for human data to support it. The single human data we show the reward rate of in Fig.5a was provided by Thomas Thierry. It comes from a dataset of 32 humans that will be reported in a publicly accessible preprint (last author: Karim Jerbi) in February 2022 according to the authors. This study used the same task design and values for task parameters as ref. 17. To assess R3's concern, we analyzed the entire dataset of 32 humans. We now show in Figure 5a, the mean and standard deviation of reward rates across these 32 subjects and have updated the caption accordingly. Note that we have included in trial duration (used the calculation of the reward rates) the mean of a random initial wait time (which was previously omitted). Accordingly, we have added the text describing T_ITI at line 788

> "It was set to 7.5 steps, the value used in \cite{Thura2014}. In practice, this time also includes a random wait time (uniformly distributed between 2 and 3 steps) at the beginning of the trial to allow the subject to set-up. We excluded this time from our analysis. Only trials in which the subject completed the trial were analyzed."

As for NHPs, we analyzed all the data from ref. 17, which performed experiments with 2 monkeys. Albeit a small sample size, our analysis showed that the two monkeys serving as independent samples differed in a way that could be captured by PGD. Given the large number of trials, the standard error bars on the estimates of their reward rates in fig.5a are too small to see.

D. It is a bit unclear to me what tasks does PGD generalize at the moment. Even though this point is mentioned in the Discussion, I think it would make sense to fit it to more proper foraging data, or to a task where it can be shown how animals learn (and fail to learn) the optimal time to invest in a decision.

Thanks for the suggestion! We saw value in our choice of task as the data we had for it provided us with both behavioural and neural data that allowed us to link these two modalities in a single model. We are unaware of available datasets for foraging tasks that contain both these modalities. We believe pursuing it falls outside of the current scope of the paper, which is already lengthy. Nevertheless, we do provide public code that can be adapted to different data sets such as that of foraging tasks. We will reiterate this point and the potential application to experimental foraging data in the discussion (line 595):

> We expect that the ideas behind PGD can also provide an explanation to the context dependence observed in response times in non-episodic tasks with varying context (e.g. in bandit tasks \cite{Fontanesi2019}) and foraging tasks.

Minor points:

- The introduction paragraph confuses the idea of spending time doing something with the idea of spending time deciding (what to do or to choose). The examples they make are more in line with the former, while the topic of urgency has to do with the latter. I suggest changing the first paragraph.

This point was also made by R2. We have rewritten the introduction to this effect. The connection between the two is that the ‘something’ that is being done is deliberation, ie. deciding what to do. Please see the above response as well as text starting at line 17

- Line 147: "A foraging agent" not "An foraging agent"

This has been corrected.

- Figure 1d: I cannot distinguish the 2 colors

We have modified the colors such that they have higher contrast.
